# Supplementary material for: The Scope for Thalassemia Gene Therapy by Disruption of Aberrant Regulatory Elements
Source: J Clin Med. 2019 Nov 13;8(11):1959. doi: 10.3390/jcm8111959 (PMC6912506; doi:10.3390/jcm8111959)
Supplement: Supplementary file 1 [file jcm-08-01959-s001.pdf]

Journal of
Clinical Medicine

MDPI

Contents

1. Supplementary Figures

Figure S1. Flow cytometry of electroporated MEL cell line.

Figure S2. Transfection and targeted disruption efficiencies in MEL *HBB*^{IVS} bulk cells.

Figure S3. Characterization of TALEN- and RGN-induced indels in MEL-*HBB*^{IVS} as bacterial clones.

Figure S4. Comparison of TALEN data in CD34+ and MEL cells

Figure S5. Schematic workflow of DARE target and nuclease compilation for new target loci.

2. Supplementary Tables

Table S1. Known β-thalassemia mutations passing the initial filter criteria for analysis.

3. References

1. Supplementary Figures

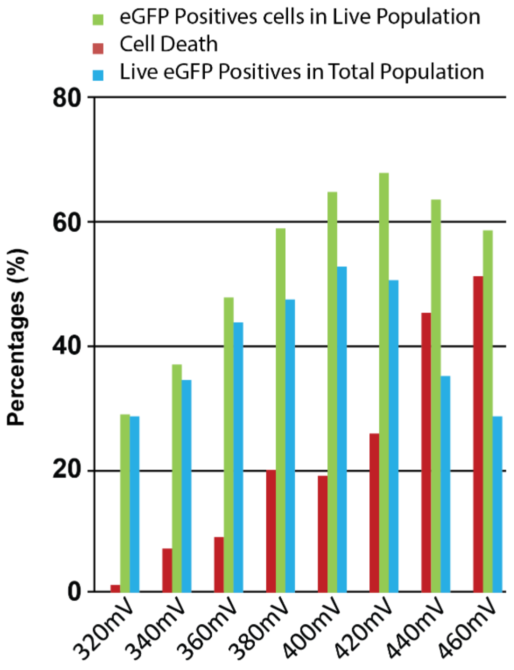

Figure S1. Flow cytometry of electroporated MEL cell line. Optimization of plasmid electroporation conditions in MEL cells. MEL cells were analyzed by flow cytometry 48 h after delivering the GFP reporter plasmid by electroporation, using a range of voltages and constant 1050 μF capacitance. Cell death (% SYTOX Red positives) was background-corrected for that of the non-electroporated negative control (≈20%, not shown). Optimal electroporation conditions (highlighted in yellow) were those with the highest percentage of live GFP positives in the total population (400 mV and 1050 μF).

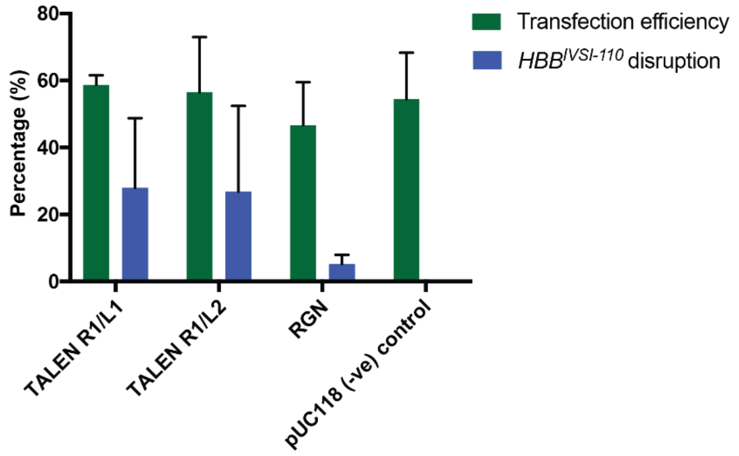

Figure S2. Transfection and targeted disruption efficiencies in MEL HBB^{IVS} bulk cells. Transfection efficiency is shown as the average percentage of GFP positives measured by flow cytometry 48 h post-electroporation (green bars), and percentages of $HBB^{IVSI-110(G>A)}$-targeted disruption on day 5 post-electroporation of (recovered) MEL HBB^{IVS} bulk populations as measured by the T7E1 assay (blue bars). All displayed data comprised the average values of biological triplicates (n = 3; ±SD).

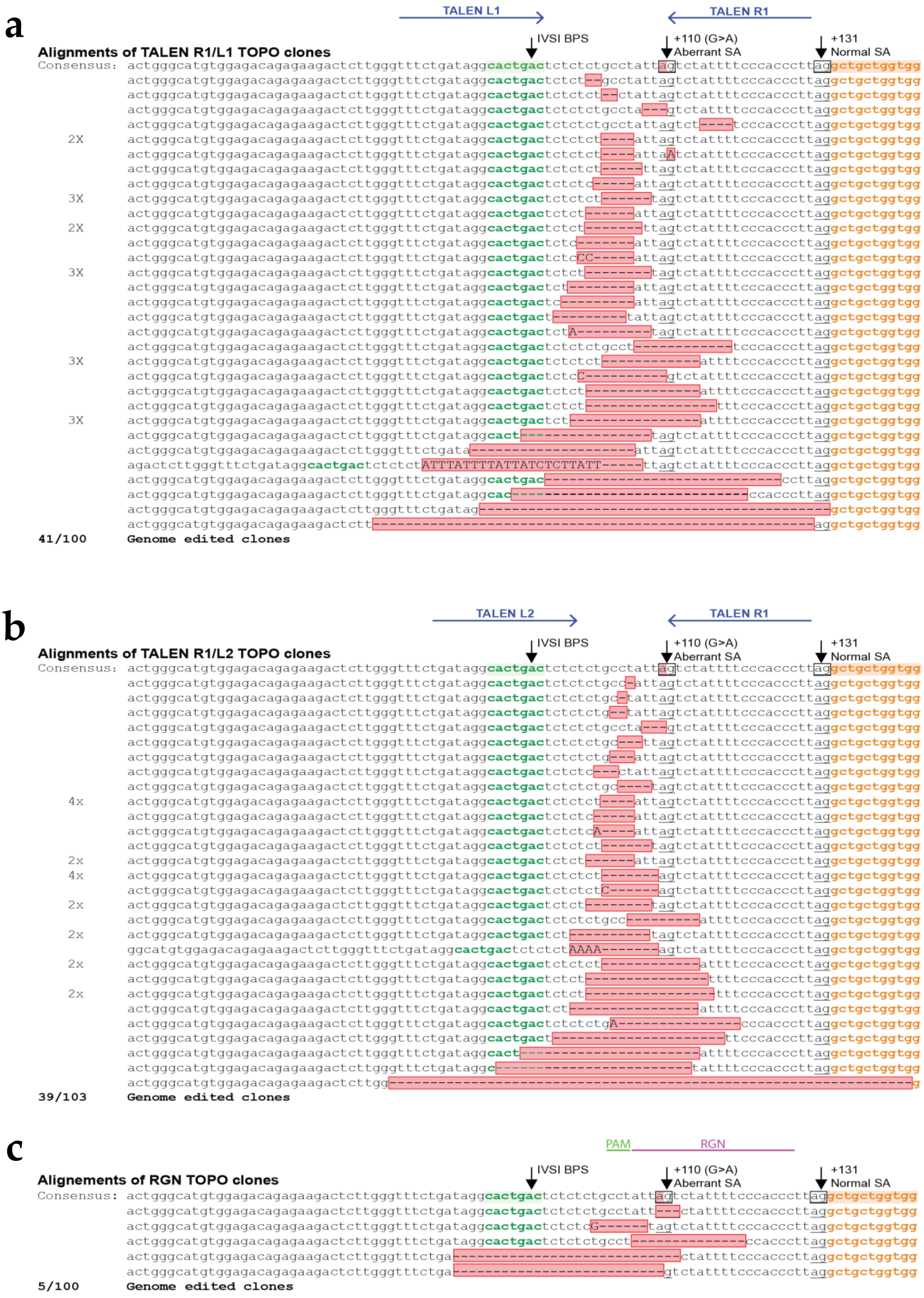

Figure S3. Characterization of TALEN- and RGN-induced indels in MEL-*HBB*^{IVS} as bacterial clones. Alignments of TOPO clones holding disrupted *HBB*$^{IVSI\text{-}110(G>A)}$ amplicons derived from HBB TALEN R1/L1 (**a**), TALEN R1/L2 (**b**) and RGN (**c**) genome-modified bulk MEL-*HBB*^{IVS} populations. TOPO clones were aligned primarily based on the size of the indel and secondarily based on indel proximity

to the normal splice acceptor site (+131 Normal SA). Intron 1 is shown unshaded, the intron-1 branchpoint site (IVSI BPS) in green, exon 2 in orange, the $HBB^{IVSI\text{-}110(G>A)}$ mutation in red, and the NHEJ-induced indels in pink. Aberrant (+110 (G>A) Aberrant SA) and normal (+131 Normal SA) splice acceptor sites are underlined (ag) sequences on the consensus sequence. Combined editing events of insertions (upper case) and deletions are shown. Binding sites of TALEN monomers are shown as blue arrows (A and B) and RGN gRNA and PAM sequence as purple and green lines (C) above each consensus sequence.

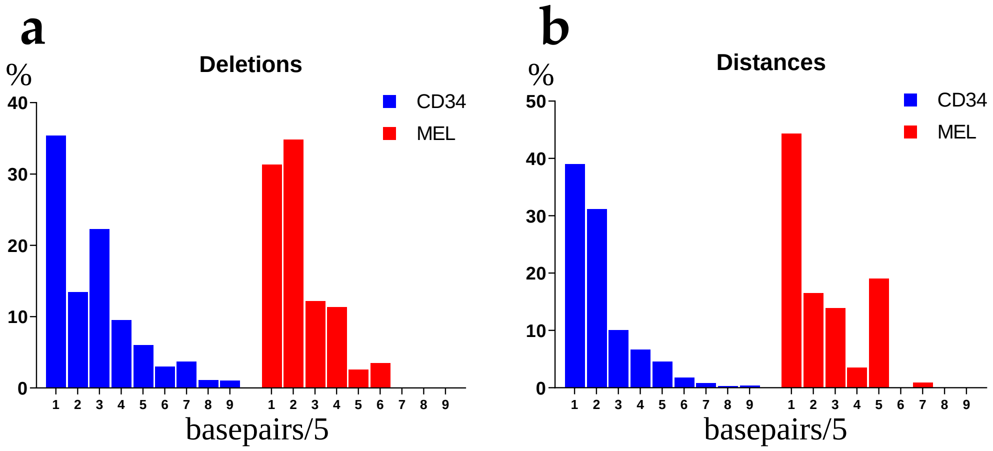

Figure S4. Comparison of TALEN data in CD34+ and MEL cells. Frequencies (**a**) of deletion sizes of the original $HBB^{IVSI\text{-}110(G>A)}$ on-target sequence and (**b**) the greatest distance of deletions from the predicted cleavage site are compared for $HBB^{IVSI\text{-}110(G>A)}$–homozygous $CD34^{+}$ cells [1] and MEL-HBBIVS cells (this study) after categorization in bin sizes of five base pairs. The Spearman constant for the correlation of the categorized data is 0.865 (*p* value of correlation: 0.007) for (**a**) and 0.848 (*p* value of correlation: 0.005) for (**b**). The Spearman constant for the underlying uncategorized data is 0.786 (*p* value of correlation: 3.196×10^{-12}) for (**a**) and 0.745 (*P* value of correlation: 1.580×10^{-10}) for (**b**).

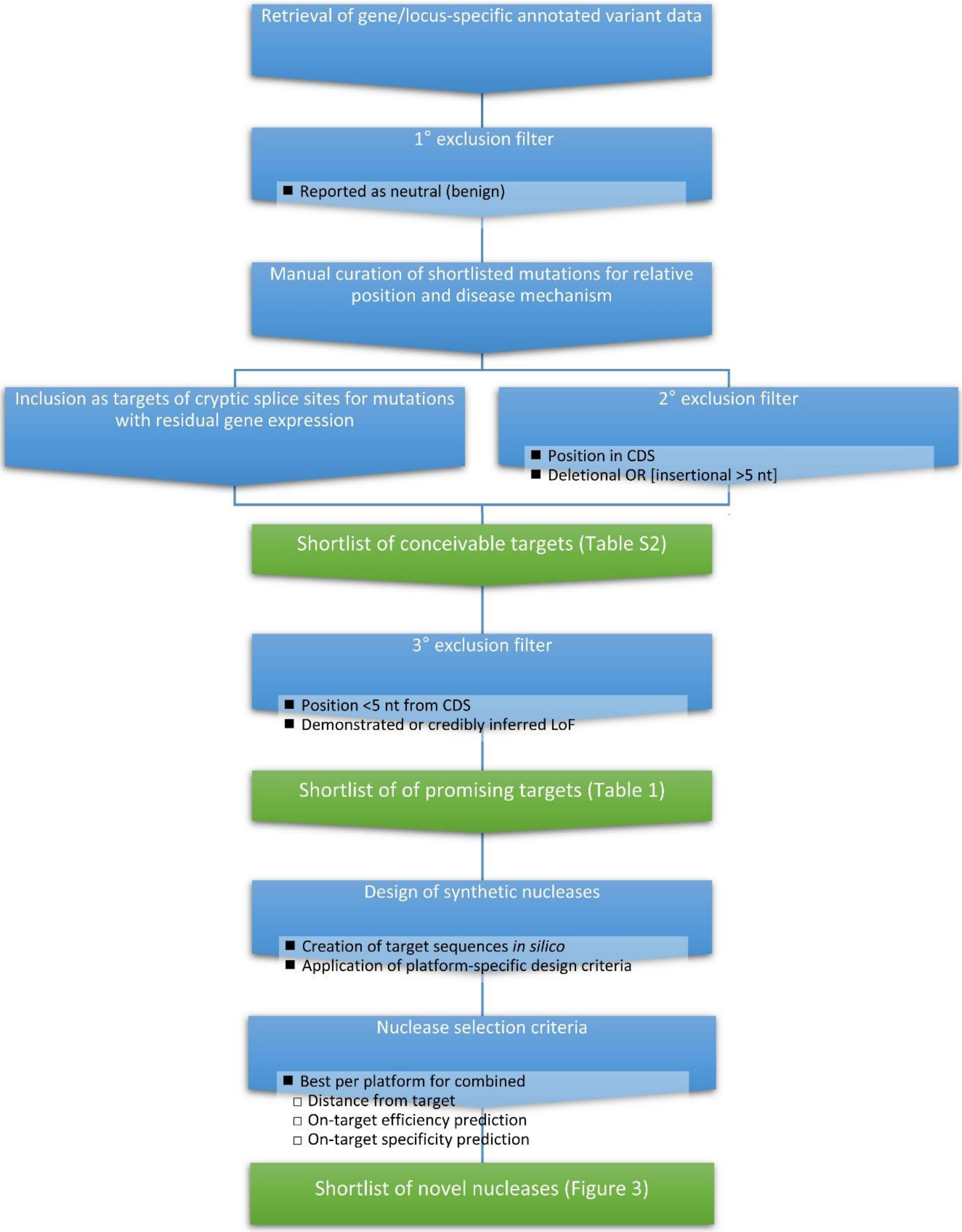

Figure S5. Schematic workflow of the DARE target and nuclease compilation for new target loci. 2° and 3° exclusion filters can be combined, where a list of conceivable targets is not of interest. CDS—coding sequence; LoF—loss of function; nt—nucleotide.

2. Supplementary Tables

Table S1. Known β-thalassemia mutations passing initial filter criteria for analysis.

IthaID[1]	Common Name	HGVS Name	Type of mutation	Region	Exon⇥[2]	References
3081[3]	-223 T>C	HBB:c.-273T>C	Likely RE[4] LoF[5]	Upstream promoter	273 nt[6]	[2]
1	-190 G>A	HBB:c.-240G>A	Likely RE LoF	Upstream promoter	240 nt	[3]
2	-102 C>A	HBB:c.-152C>A	RE LoF	CACCC box, distal	152 nt	[4]
3	-101 C>T	HBB:c.-151C>T	RE LoF	CACCC box, distal	151 nt	[5]
4	-101 C>G	HBB:c.-151C>G	RE LoF	CACCC box, distal	151 nt	[6]
3059	-98 T>A	HBB:c.-148T>A	Possible RE LoF	near CACCC boxes	148 nt	[7]
5	-93 C>G	HBB:c.-143C>G	RE LoF	near CACCC boxes	143 nt	ITHANET[7]
6	-92 C>T	HBB:c.-142C>T	RE LoF	near CACCC boxes	142 nt	[8]
7	-90 C>T	HBB:c.-140C>T	RE LoF	CACCC box, proximal	140 nt	[7]
3224	-90 C>G	HBB:c.-140C>G	RE LoF	CACCC box, proximal	140 nt	[9]
8	-88 C>T	HBB:c.-138C>T	RE LoF	CACCC box, proximal	138 nt	[7]
9	-88 C>A	HBB:c.-138C>A	RE LoF	CACCC box, proximal	138 nt	[10]
2178	-88 C>G	HBB:c.-138C>G	RE LoF	CACCC box, proximal	138 nt	[11]
10	-87 C>G	HBB:c.-137C>G	RE LoF	CACCC box, proximal	137 nt	[12]
11	-87 C>T	HBB:c.-137C>T	RE LoF	CACCC box, proximal	137 nt	[13]
12	-87 C>A	HBB:c.-137C>A	RE LoF	CACCC box, proximal	137 nt	[14]
13	-86 C>G	HBB:c.-136C>G	RE LoF	CACCC box, proximal	136 nt	[15]
14	-86 C>A	HBB:c.-136C>A	RE LoF	CACCC box, proximal	136 nt	[13]
3077	-83 G>A	HBB:c.-133G>A	Possible RE LoF	near CACCC & CCAAT	133 nt	[16]
3069	-77 G>C	HBB:c.-127G>C	Possible RE LoF	near CACCC & CCAAT	127 nt	[17]
3386	-76 C>A	HBB:c.-126C>A	RE LoF	CCAAT box	126 nt	[18]
15	-73 A>T	HBB:c.-123A>T	RE LoF	CCAAT box	123 nt	[19]
2997	-72 T>A	HBB:c.-122T>A	RE LoF	CCAAT box	122 nt	[20]
2171	-71 C>T	HBB:c.-121C>T	Likely RE LoF	DRE[8]	121 nt	[21]
3043	-71 C>T	HBB:c.-121C>T	Likely RE LoF	DRE	121 nt	[11]
16	-56 G>C	HBB:c.-106G>C	Likely RE LoF	DRE	106 nt	[3]
17	-50 G>A	HBB:c.-100G>A	Likely RE LoF	DRE	100 nt	[22]
3060	-42 C>G	HBB:c.-92C>G	Likely RE LoF	DRE	92 nt	[7]
2172	-41 A>T	HBB:c.-91A>C	Likely RE LoF	DRE	91 nt	[23]
18	-32 C>A	HBB:c.-82C>A	Likely RE LoF	DRE	82 nt	[24]
19	-32 C>T	HBB:c.-82C>T	Likely RE LoF	DRE	82 nt	[25]
20	-31 A>G	HBB:c.-81A>G	RE LoF	TATA (ATAAA) box	81 nt	[26]
21	-31 A>C	HBB:c.-81A>C	RE LoF	TATA (ATAAA) box	81 nt	[27]
22	-30 T>A	HBB:c.-80T>A	RE LoF	TATA (ATAAA) box	80 nt	[28]
23	-30 T>C	HBB:c.-80T>C	RE LoF	TATA (ATAAA) box	80 nt	[29]
2179	-30 T>G	HBB:c.-80T>G	RE LoF	TATA (ATAAA) box	80 nt	[11]
25	-29 A>G	HBB:c.-79A>G	RE LoF	TATA (ATAAA) box	79 nt	[30]
26	-29 A>C	HBB:c.-79A>C	RE LoF	TATA (ATAAA) box	79 nt	[31]
28	-28 A>C	HBB:c.-78A>C	RE LoF	TATA (ATAAA) box	78 nt	[32]
29	-28 A>G	HBB:c.-78A>G	RE LoF	TATA (ATAAA) box	78 nt	[33]
30	-27 A>T	HBB:c.-77A>T	Likely RE LoF	near TATA (ATAAA)	77 nt	[34]
2175	-26 A>C	HBB:c.-76A>C	Likely RE LoF	near TATA (ATAAA)	76 nt	[23]
32	-25 G>C	HBB:c.-75G>C	Likely RE LoF	near TATA (ATAAA)	75 nt	[25]
2565	-25 G>T	HBB:c.-75G>T	Likely RE LoF	near TATA (ATAAA)	75 nt	[35]
34	CAP +1 A>C	HBB:c.-50A>C	LoF	5′ UTR	50 nt	[36]
3464	CAP +3 A>T	HBB:c.-48A>T	LoF	CAP initiator element	48 nt	[37]
35	CAP +8 C>T	HBB:c.-43C>T	Likely LoF	5′ UTR	43 nt	[38]
36	CAP +10 -T	HBB:c.-41delT	Mild LoF	5′ UTR	41 nt	[39]
2494	CAP +16 A>G	HBB:c.-35A>G	Mild LoF	5′ UTR	35 nt	[40]
3345	CAP +22 G>T	HBB:c.-29G>T	Mild LoF	5′ UTR	29 nt	[41]
38	CAP +22 G>A	HBB:c.-29G>A	Mild LoF	5′ UTR	29 nt	[42]
2536	CAP +30 T>A	HBB:c.-21T>A	Mild LoF	5′ UTR	21 nt	[43]
39	CAP +33 C>G	HBB:c.-18C>G	Mild LoF	5′ UTR	18 nt	[44]
2176	CAP +39 C>T	HBB:c.-12C>T	Mild LoF	5′ UTR	12 nt	[45]
40	CAP +40 to +43 (-AAAC)	HBB:c.-11_-8delAAAC	LoF	5′ UTR	8 nt	[46]
41	CAP +45 (G>C)	HBB:c.-6G>C	Mild LoF	5′ UTR, Kozak sequence	6 nt	[47]
107	IVS I-5 G>A	HBB:c.92+5G>C	Activation of cSD[9]; partial SD LoF	SD[10]-proximal	5 nt	[12,48]

111	IVS I-6 T>C	HBB:c.92+6T>C	Activation of cSD; partial SD LoF	SD-proximal	6 nt	[49]
112	IVS I-7 A>T	HBB:c.92+7A>T	Unknown	SD-proximal	7 nt	ITHANET[7]
3276	IVS I-7 A>G	HBB:c.92+7A>G	Unknown	SD-proximal	7 nt	ITHANET[7]
3445	IVSI-13G	HBB:c.92+13	Potential target	cSD activated by IthaID	13 nt	[12]
113	IVS I-110 G>A	HBB:c.93-21G>A	**Confirmed target; GG>GA (aSA)**[11]	aSA	21 nt	[1,50]
3008	IVS I-115 A>T	HBB:c.93-16A>T	AT>TT (effect unclear)	Intronic	16 nt	[51]
114	IVS I-116 T>G	HBB:c.93-15T>G	TT>GT (potential aSD)[12]	Intronic	15 nt	[52]
115	IVS I-128 T>G	HBB:c.93-3T>G	SS LoF	3' pyrimidine run	3 nt	[53]
116	IVS I-129 A>C	HBB:c.93-2A>C	SS LoF	SA[13]	2 nt	[54]
117	IVS I-129 A>G	HBB:c.93-2A>G	SS LoF	SA	2 nt	[55]
118	IVS I-130 G>C	HBB:c.93-1G>C	SS LoF	SA	1 nt	[56]
119/120	IVS I-130 G>A	HBB:c.93-1G>A	SS LoF	SA	1 nt	[7,57–59]
200	IVS II-1 G>A	HBB:c.315+1G>A	SS LoF	SD	1 nt	[60,61]
201	IVS II-1 G>C	HBB:c.315+1G>C	SS LoF	SD	1 nt	[62]
202	IVS II-1 G>T	HBB:c.315+1G>T	SS LoF	SD	1 nt	ITHANET[7]
203	IVS II-2 T>C	HBB:c.315+2T>C	SS LoF	SD	2 nt	[63]
204	IVS II-2 T>A	HBB:c.315+2T>A	SS LoF	SD	2 nt	ITHANET[7]
3226	IVS II-2 T>G	HBB:c.315+2T>G	SS LoF	SD	2 nt	[9]
208	IVS II-5 G>C	HBB:c.315+5G>C	Activation of cSD; partial LoF	Intronic	5 nt	[64,65]
3446	IVS II-579G	HBB:c.316-272	cSA[14] activated by IthaID 214	Intron	270 nt	[12]
210	IVS II-613 C>T	HBB:c.316-238C>T	Activation of cryptic splice site	Intron	238 nt	[66]
211	IVS II-654 C>T	HBB:c.316-197C>T	**Confirmed target; GC>GT (aSD)**	Intron	197 nt	[50,67]
212	IVS II-705 T>G	HBB:c.316-146T>G	Activation of cryptic splice site	Intron	146 nt	[68,69]
213	IVS II-726 A>G	HBB:c.316-125A>G	Likely block of RNA processing	Intron	125 nt	[70]
214	IVS II-745 C>G	HBB:c.316-106C>G	aSD, activating cSA IthaID3446	Intron	106 nt	[12]
215	IVS II-761 A>G	HBB:c.316-90A>G	AT>GT (potential aSD	Intron	90 nt	ITHANET[7]
2183	IVS II-781 C>G	HBB:c.316-70C>G	CT>GT (potential aSD)	Intron	70 nt	[11]
216	IVS II-815 C>T	HBB:c.316-36C>T	CT>GT (potential aSD)	Intron	36 nt	[71]
217	IVS II-837 T>G	HBB:c.316-14T>G	AT>AG (potential aSA)	Intron	14 nt	[72]
218	IVS II-843 T>G	HBB:c.316-8T>G	LoF	3' pyrimidine run	8 nt	[73]
219	IVS II-844 C>A	HBB:c.316-7C>A	Presumed LoF, very mild	3' pyrimidine run	7 nt	[74]
220	IVS II-844 C>G	HBB:c.316-7C>G	Presumed LoF, very mild	3' pyrimidine run	7 nt	[75,76]
221	IVS II-848 C>A	HBB:c.316-3C>A	LoF	3' pyrimidine run	3 nt	[53,77]
222	IVS II-848 C>G	HBB:c.316-3C>G	LoF	3' pyrimidine run	3 nt	[78]
3045	IVS II-848 C>T	HBB: c.316-3C>T	Presumed LoF	3' pyrimidine run	3 nt	[11]
267	Terminal CD +6 C>G [CAP +1480]	HBB:c.*6C>G	Mild LoF	3′ UTR	6 nt	[79]
2177	Terminal CD +32	HBB:*32A>C	Presumed LoF	3′ UTR	32 nt	[45]
268	Terminal CD +47	HBB:c.*47C>G	Presumed LoF	3′ UTR	47 nt	ITHANET[7]
3443	Cap +1570 (T>C)	HBB:c*96T>C	Presumed LoF, very mild	3′ UTR	96 nt	[80]
278	Poly A (-AATAA) AATAAA> -----	HBB:c.*108_*112delAATAA	LoF	poly(A) signal	108 nt	[81]
270	Poly A (A>C) AATAAA>CATAAA	HBB:c.*108A>C	Mild LoF	poly(A) signal	108 nt	[82]
271	Poly A (A>G) AATAAA>GAT	HBB:c.*108A>G	LoF	poly(A) signal	108 nt	[83]
277	Poly A -AT	HBB:c.*109_*110delAT | HBB:c.*110_*111delTA	LoF	poly(A) signal	109 nt	[84]
272	Poly A (T>C) AATAAA>AACAAA	HBB:c.*110T>C	Mild LoF	poly(A) signal	110 nt	[85]
273	Poly A (T>A) AATAAA>AAAAAA	HBB:c.*110A>C	Mild LoF	poly(A) signal	110 nt	[86]
3046	Poly(A) AATAAA>AAT--A	HBB:c.*111_*112delAA	Mild LoF	poly(A) signal	111 nt	[87]
274	Poly A (A>G) AATAAA>AATGAA	HBB:c.*111A>G	Mild LoF	poly(A) signal	111 nt	[88]

2198	Poly A (A>T) AATAAA>AATATA	HBB:c.*112A>T	Mild LoF	poly(A) signal	112 nt	[89]
275	Poly A (A>G) AATAAA>AATAGA	HBB:c.*112A>G	Mild LoF	poly(A) signal	112 nt	[88]
276	Poly A (A>G) AATAAA>AATAAG	HBB:c.*113A>G	Mild LoF	poly(A) signal	113 nt	[90]
2564	3'UTR +1592	HBB:c.*118A>G	Very mild or benign	Conserved +1592 nt in 3'	1592 nt	[91]
2463	3'UTR +101 G>C	HBB:c.*233G>C	Very mild or benign	3' UTR-adjacent	101 nt	[92]

Fill color for "*Type of mutation*" indicates likely suitability for DARE, based on *Region*, severity and level of characterization. Absence of fill color ––unsuitable for DARE; orange fill color—likely unsuitable for DARE; yellow fill color—possibly suitable for DARE; green fill color—likely or proven suitable for DARE and included in Table 1 of the manuscript.

[1] Nucleotide-specific target ID from ITHANET (www.ithanet.eu); [2] Distance of the target from the nearest exon; [3] IthaID3083 was the only *HBB* mutation detected in heterozygosity in a β-thalassemic patient. The second mutation is presumed to have escaped detection. IthaID3083 would be a potential target if it turned out to be dominant after all; [4] RE—response element; [5] LoF—loss of function; [6] nt—nucleotide; [7] Unpublished data retrieved from the ITHANET Portal [93]; [8] DRE—direct repeat element; [9] cSD—cryptic splice donor; [10] SD—splice donor; [11] aSA—aberrant splice acceptor; [12] aSD—aberrant splice donor; [13] SA—splice acceptor; [14] cSA—cryptic splice acceptor

References

1. Patsali, P.; Turchiano, G.; Papasavva, P.; Romito, M.; Loucari, C.C.; Stephanou, C.; Christou, S.; Sitarou, M.; Mussolino, C.; Cornu, T.I.; et al. Correction of IVS I-110(G>A) β-thalassemia by CRISPR/Cas- and TALEN-mediated disruption of aberrant regulatory elements in human hematopoietic stem and progenitor cells. *Haematologica* **2019**, Epub.
2. Nagar, R.; Sinha, S.; Raman, R. Genotype-phenotype correlation and report of novel mutations in β-globin gene in thalassemia patients. *Blood Cells. Mol. Dis.* **2015**, *55*, 10–4.
3. Agouti, I.; Bennani, M.; Nezri, M.; Levy, N.; Badens, C. Beta-thalassemia intermedia due to two novel mutations in the promoter region of the beta-globin gene. *Eur J Haematol* **2008**, *80*, 346–350.
4. Aguilar-Martinez, P.; Jourdan, E.; Brun, S.; Cunat, S.; Giansily-Blaizot, M.; Pissard, S.; Schved, J.-F. A novel mutation of the beta-globin gene promoter (-102 C>A) and pitfalls in family screening. *Am. J. Hematol.* **2007**, *82*, 1088–90.
5. Maragoudaki, E.; Kanavakis, E.; Traeger-Synodinos, J.; Vrettou, C.; Tzetis, M.; Metaxotou-Mavrommati, A.; Kattamis, C. Molecular, haematological and clinical studies of the -101 C --> T substitution of the beta-globin gene promoter in 25 beta-thalassaemia intermedia patients and 45 heterozygotes. *Br J Haematol* **1999**, *107*, 699–706.
6. Moi, P.; Faa, V.; Marini, M.G.; Asunis, I.; Ibba, G.; Cao, A.; Rosatelli, M.C. A novel silent beta-thalassemia mutation in the distal CACCC box affects the binding and responsiveness to EKLF. *Br. J. Haematol.* **2004**, *126*, 881–884.
7. Yasmeen, H.; Toma, S.; Killeen, N.; Hasnain, S.; Foroni, L. The molecular characterization of Beta globin gene in thalassemia patients reveals rare and a novel mutations in Pakistani population. *Eur. J. Med. Genet.* **2016**, *59*, 355–62.
8. Rosatelli, M.C.; Faà, V.; Meloni, A.; Fiorenza, F.; Galanello, R.; Gasperini, D.; Amendola, G.; Cao, A. A promoter mutation, C-->T at position -92, leading to silent beta-thalassaemia. *Br. J. Haematol.* **1995**, *90*, 483–5.
9. Rizo-de-la-Torre, L.C.; Ibarra, B.; Sánchez-López, J.Y.; Magaña-Torres, M.T.; Rentería-López, V.M.; Perea-Díaz, F.J. Three novel HBB mutations, c.-140C>G (-90 C>G), c.237_256delGGACAACCTCAAGGGCACCT (FS Cd 78/85 -20 bp), and c.315+2T>G (IVS2:2 T>G). Update of the mutational spectrum of β-Thalassemia in Mexican mestizo patients. *Int. J. Lab. Hematol.* **2017**, *39*, 539–545.
10. Rund, D.; Cohen, T.; Filon, D.; Dowling, C.E.; Warren, T.C.; Barak, I.; Rachmilewitz, E.; Kazazian, H.H.; Oppenheim, A. Evolution of a genetic disease in an ethnic isolate: beta-thalassemia in the Jews of Kurdistan. *Proc. Natl. Acad. Sci. U. S. A.* **1991**, *88*, 310–4.
11. Henderson, S.J.; Timbs, A.T.; McCarthy, J.; Gallienne, A.E.; Proven, M.; Rugless, M.J.; Lopez, H.; Eglinton, J.; Dziedzic, D.; Beardsall, M.; et al. Ten Years of Routine α- and β-Globin Gene Sequencing in UK Hemoglobinopathy Referrals Reveals 60 Novel Mutations. *Hemoglobin* **2016**, *40*, 75–84.
12. Treisman, R.; Orkin, S.H.; Maniatis, T. Specific transcription and RNA splicing defects in five cloned beta-thalassaemia genes. *Nature* **1983**, *302*, 591–596.
13. Meloni, A.; Rosatelli, M.C.; Faà, V.; Sardu, R.; Saba, L.; Murru, S.; Sciarratta, G. V.; Baldi, M.; Tannoia, N.; Vitucci, A.; et al. Promoter mutations producing mild β-thalassaemia in the Italian population. *Br. J. Haematol.* **1992**, *80*, 222–226.
14. Coleman, M.B.; Steinberg, M.H.; Harrell, A.H.; Plonczynski, M.W.; Walker, A.M.; Adams, J.G. The -87 (C→A) β^{+}-Thalassemia Mutation in a Black Family. *Hemoglobin* **1992**, *16*, 399–401.
15. Thein, S.L.; Winichagoon, P.; Hesketh, C.; Best, S.; Fucharoen, S.; Wasi, P.; Weatherall, D.J. The molecular basis of beta-thalassemia in Thailand: application to prenatal diagnosis. *Am. J. Hum. Genet.* **1990**, *47*, 369–75.
16. Cadet, E.; Foulon, K.; Claisse, J.-F.; Rochette, J. First identification of a point mutation at position -83 (G>A) of the beta-globin gene promoter. *Hemoglobin* **2009**, *33*, 274–8.
17. Bilgen, T.; Canatan, D.; Delibas, S.; Keser, I. A Novel Mutation in the Promoter Region of the β-Globin Gene: HBB: c.-127G > C. *Hemoglobin* **2016**, *40*, 280–2.
18. Refaldi, C.; Mocellin, M.C.; Cappellini, M.D. Gene symbol: HBB. *Hum. Genet.* **2007**, *121*, 298.
19. Chen, X.-W.; Mo, Q.-H.; Li, Q.; Zeng, R.; Xu, X.-M. A novel mutation of −73(A→T) in the CCAAT box of the β-globin gene identified in a patient with the mild β-thalassemia intermedia. *Ann. Hematol.* **2007**, *86*, 653–657.

20. Ma, Q.; Abel, K.; Sripichai, O.; Whitacre, J.; Angkachatchai, V.; Makarasara, W.; Winichagoon, P.; Fucharoen, S.; Braun, A.; Farrer, L.A. Beta-globin gene cluster polymorphisms are strongly associated with severity of HbE/beta(0)-thalassemia. *Clin Genet* **2007**, *72*, 497–505.
21. Al Zadjali, S.; Wali, Y.; Al Lawatiya, F.; Gravell, D.; Alkindi, S.; Al Falahi, K.; Krishnamoorthy, R.; Daar, S. The β-globin promoter -71 C>T mutation is a β+ thalassemic allele. *Eur. J. Haematol.* **2011**, *87*, 457–60.
22. Li, D.-Z.; Liao, C.; Xie, X.-M.; Zhou, J.-Y. A novel mutation of -50 (G-->A) in the direct repeat element of the beta-globin gene identified in a patient with severe beta-thalassemia. *Ann. Hematol.* **2009**, *88*, 1149–50.
23. Salehi, R.; Fisher, C.A.; Bignell, P.A.; Eslami, G.; Old, J.M. Identification of three novel mutations [-41 (A>C), codon 24 (-G), and IVS-I-109 (-T)], in a study of beta-thalassemia alleles in the Isfahan region of Iran. *Hemoglobin* **2010**, *34*, 115–20.
24. Lin, C.-K.; Lin, J.-S.; Jiang, M.-L. Letter to the editor: Iron absorption is increased in hemoglobin H diseases. *Am. J. Hematol.* **1992**, *40*, 74–75.
25. Eng, B.; Walker, L.; Nakamura, L.M.; Hoppe, C.; Azimi, M.; Lee, H.; Waye, J.S. Three new beta-globin gene promoter mutations identified through newborn screening. *Hemoglobin* **2007**, *31*, 129–34.
26. Takihara, Y.; Nakamura, T.; Yamada, H.; Takagi, Y.; Fukumaki, Y. A novel mutation in the TATA box in a Japanese patient with beta +-thalassemia. *Blood* **1986**, *67*, 547–50.
27. Huisman, T.H.J. Compound Heterozygosity for Hb S and the Hybrid Hbs Lepore, P-Nilotic, and Kenya; Comparison of Hematological and Hemoglobin Composition Data. *Hemoglobin* **1997**, *21*, 249–257.
28. Fei, Y.J.; Stoming, T.A.; Efremov, G.D.; Efremov, D.G.; Battacharia, R.; Gonzalez-Redondo, J.M.; Altay, C.; Gurgey, A.; Huisman, T.H. Beta-thalassemia due to a T----A mutation within the ATA box. *Biochem. Biophys. Res. Commun.* **1988**, *153*, 741–7.
29. Cai, S.P.; Zhang, J.Z.; Doherty, M.; Kan, Y.W. A new TATA box mutation detected at prenatal diagnosis for β-thalassemia. *Am. J. Hum. Genet.* **1989**, *45*, 112–114.
30. Antonarakis, S.E.; Irkin, S.H.; Cheng, T.C.; Scott, A.F.; Sexton, J.P.; Trusko, S.P.; Charache, S.; Kazazian, H.H. beta-Thalassemia in American Blacks: novel mutations in the "TATA" box and an acceptor splice site. *Proc. Natl. Acad. Sci.* **1984**, *81*, 1154–1158.
31. Sadiq, M.F.; Eigel, A.; Horst, J. Spectrum of β-thalassemia in Jordan: Identification of two novel mutations. *Am. J. Hematol.* **2001**, *68*, 16–22.
32. Poncz, M.; Ballantine, M.; Solowiejczyk, D.; Barak, I.; Schwartz, E.; Surrey, S. beta-Thalassemia in a Kurdish Jew. Single base changes in the T-A-T-A box. *J. Biol. Chem.* **1982**, *257*, 5994–6.
33. Orkin, S.H.; Sexton, J.P.; Cheng, T.; Goff, S.C.; Giardina, P.J. V.; Joseph, I.L.; Hazazian, H.H. ATA box transcription mutation in *β* -thalassemia. *Nucleic Acids Res.* **1983**, *11*, 4727–4734.
34. al., B. et Characterization of a New Polymorphism, IVS-I-108 (T→C), and a New β-Thalassemia Mutation, -27 (A→T), Discovered in the Course of a Prenatal Diagnosis. *Hemoglobin* **1999**, *23*, 339–344.
35. Li, Z.; Li, L.; Yao, Y.; Li, N.; Li, Y.; Zhang, Z.; Yan, F.; Qiu, H.; Wu, C.; Zhang, Z. A novel promoter mutation (HBB: c.-75G>T) was identified as a cause of β(+)-thalassemia. *Hemoglobin* **2015**, *39*, 115–20.
36. Wong, C.; Dowling, C.E.; Saiki, R.K.; Higuchi, R.G.; Erlich, H.A.; Kazazian, H.H. Characterization of β-thalassaemia mutations using direct genomic sequencing of amplified single copy DNA. *Nature* **1987**, *330*, 384–386.
37. Letitia, D.; Rodica, T.; Xenia, F.; Daniel, C.; Florentina, V.; Marina, K.; Ciprian, T.; Lucian, G. A novel β-globin gene CAP site mutation in association with the 92+1G>A mutation was found in a thalassemia intermedia patient. *Rom. Biotechnol. Lett.* **2011**, *16*, 6042–6047.
38. Ma, S.K.; Ha, S.Y.; Chan, A.Y.Y.; Chan, G.C.F.; Lau, Y.L.; Chan, L.C. Two Novel β-Thalassemia Alleles in the Chinese: the IVS-II-2 (−T) and Nucleotide +8 (C→T) β-Globin Gene Mutations. *Hemoglobin* **2000**, *24*, 327–332.
39. Athanassiadou, A.; Papachatzopoulou, A.; Zoumbos, N.; Maniatis, G.M.; Gibbs, R. A novel beta-thalassaemia mutation in the 5′ untranslated region of the beta-globin gene. *Br. J. Haematol.* **1994**, *88*, 307–10.
40. Vinciguerra, M.; Passarello, C.; Leto, F.; Cassarà, F.; Cannata, M.; Maggio, A.; Giambona, A. Identification of three new nucleotide substitutions in the β-globin gene: laboratoristic approach and impact on genetic counselling for beta-thalassaemia. *Eur. J. Haematol.* **2014**, *92*, 444–9.
41. Vinciguerra, M.; Passarello, C.; Cassarà, F.; Leto, F.; Cannata, M.; Calvaruso, G.; Renda, D.; Maggio, A.; Giambona, A. Coheredity of a new silent mutation: c.-29G>T, with a severe β-thal mutation in a patient with β-thalassemia intermediate. *Int. J. Lab. Hematol.* **2018**, *40*, e17–e20.
42. Oner, R.; Agarwal, S.; Dimovski, A.J.; Efremov, G.D.; Petkov, G.H.; Altay, C.; Gurgey, A.; Huisman, T.H.

The G----A mutation at position +22 3′ to the Cap site of the beta-globin gene as a possible cause for a beta-thalassemia. *Hemoglobin* **1991**, *15*, 67–76.

43. Aldemir, O.; Izmirli, M.; Kaya, H. The spectrum of β-thalassemia mutations in Hatay, Turkey: Reporting three new mutations. *Hemoglobin* **2014**, *38*, 325–328.
44. Ho, P.J.; Rochette, J.; Fisher, C.A.; Wonke, B.; Jarvis, M.K.; Yardumian, A.; Thein, S.L. Moderate reduction of beta-globin gene transcript by a novel mutation in the 5′ untranslated region: a study of its interaction with other genotypes in two families. *Blood* **1996**, *87*, 1170–8.
45. Chen, W.; Zhang, X.; Shang, X.; Cai, R.; Li, L.; Zhou, T.; Sun, M.; Xiong, F.; Xu, X. The molecular basis of beta-thalassemia intermedia in southern China: Genotypic heterogeneity and phenotypic diversity. *BMC Med. Genet.* **2010**, *11*.
46. Huang, S.Z.; Xu, Y.H.; Zeng, F.Y.; Wu, D.F.; Ren, Z.R.; Zeng, Y.T. A novel beta-thalassaemia mutation: deletion of 4 bp (-AAAC) in the 5′ transcriptional sequence. *Br. J. Haematol.* **1991**, *78*, 125–6.
47. De Angioletti, M.; Lacerra, G.; Sabato, V.; Carestia, C. β+45 G → C: A novel silent β-thalassaemia mutation, the first in the Kozak sequence. *Br. J. Haematol.* **2004**, *124*, 224–231.
48. Kazazian, H.H.; Orkin, S.H.; Antonarakis, S.E.; Sexton, J.P.; Boehm, C.D.; Goff, S.C.; Waber, P.G. Molecular characterization of seven beta-thalassemia mutations in Asian Indians. *EMBO J.* **1984**, *3*, 593–596.
49. Tamagnini, G.P.; Lopes, M.C.; Castanheira, M.E.; Wainscoat, J.S.; Wood, W.G. β + Thalassaemia-Portuguese type: clinical, haematological and molecular studies of a newly defined form of β thalassaemia. *Br. J. Haematol.* **2008**, *54*, 189–200.
50. Xu, S.; Luk, K.; Yao, Q.; Shen, A.H.; Zeng, J.; Wu, Y.; Luo, H.-Y.; Brendel, C.; Pinello, L.; Chui, D.H.K.; et al. Editing aberrant splice sites efficiently restores β-globin expression in β-thalassemia. *Blood* **2019**, blood-2019-01-895094.
51. Sahoo, S.S.; Biswal, S.; Dixit, M. Distinctive mutation spectrum of the HBB gene in an urban eastern Indian population. *Hemoglobin* **2014**, *38*, 33–38.
52. Metherall, J.E.; Collins, F.S.; Pan, J.; Weissman, S.M.; Forget, B.G.; J.E., M.; Collins F.S.; Pan J.; Weissman S.M.; B.G., F.; et al. Beta zero Thalassemia caused by a base substitution that creates an alternative splice acceptor site in an intron. *EMBO J.* **1986**, *5*, 2551–2557.
53. Wong, C.; Antonarakis, S.E.; Goff, S.C.; Orkin, S.H.; Forget, B.G.; Nathan, D.G.; Giardina, P.J.; Kazazian, H.H. Beta-thalassemia due to two novel nucleotide substitutions in consensus acceptor splice sequences of the beta-globin gene. *Blood* **1989**, *73*, 914–8.
54. Old, J.M.; Khan, S.N.; Verma, I.C.; Fucharoen, S.; Kleanthous, M.; Ioannou, P.; Kotea, N.; Fisher, C.A.; Riazuddin, S.; Saxena, R.; et al. A multi-center study in order to further define the molecular basis of beta-thalassemia in Thailand, Pakistan, Sri Lanka, Mauritius, Syria, and India, and to develop a simple molecular diagnostic strategy by amplification refractory mutation system-polyme. *Hemoglobin* **2001**, *25*, 397–407.
55. Vetter, B.; Schwarz, C.; Kohne, E.; Kulozik, A.E. Beta-thalassaemia in the immigrant and non-immigrant German populations. *Br. J. Haematol.* **1997**, *97*, 266–72.
56. Yamamoto, K.; Yamamoto, K.; Hattori, Y.; Yamashiro, Y.; Hoshitani, M.; Morishita, M.; Ohba, Y.; Katahira, H.; Karasawa, M.; Omine, M. Two beta-thalassemia mutations in Japan: codon 121 (GAA----TAA) and IVS-I-130 (G----C). *Hemoglobin* **1992**, *16*, 295–302.
57. al., D. et A New β-Thalassemia Mutation Produced by a Single Nucleotide Substitution in the Conserved Dinucleotide Sequence of the IVS-I Consensus Acceptor Site (Ag→AA). *Hemoglobin* **1990**, *14*, 431–440.
58. Oner, R.; Altay, C.; Gurgey, A.; Aksoy, M.; Kilinç, Y.; Stoming, T.A.; Reese, A.L.; Kutlar, A.; Kutlar, F.; Huisman, T.H. Beta-thalassemia in Turkey. *Hemoglobin* **1990**, *14*, 1–13.
59. el-Kalla, S.; Mathews, A.R. A significant beta-thalassemia heterogeneity in the United Arab Emirates. *Hemoglobin* **1997**, *21*, 237–47.
60. Baird, M.; Driscoll, C.; Schreiner, H.; Sciarratta, G. V; Sansone, G.; Niazi, G.; Ramirez, F.; Bank, A.; al., B. et A nucleotide change at a splice junction in the human beta-globin gene is associated with beta zero-thalassemia. *Proc. Natl. Acad. Sci. U. S. A.* **1981**, *78*, 4218–4221.
61. Treisman, R.; Proudfoot, N.J.; Shander, M.; Maniatis, T. A single-base change at a splice site in a ??0-thalassemic gene causes abnormal RNA splicing. *Cell* **1982**, *29*, 903–911.
62. Nozari, G.; Rahbar, S.; Golshaiyzan, A.; Rahmanzadeh, S. Molecular analyses of beta-thalassemia in Iran. *Hemoglobin* **1995**, *19*, 425–31.
63. Frischknecht, H.; Dutly, F.; Walker, L.; Nakamura-Garrett, L.M.; Eng, B.; Waye, J.S. Three new beta-thalassemia mutations with varying degrees of severity. *Hemoglobin* **2009**, *33*, 220–5.

64. Jiang, N.H.; Liang, S.; Su, C.; Nechtman, J.F.; Stoming, T.A. A novel beta-thalassemia mutation [IVS-II-5 (G-->C)] in a Chinese family from Guangxi Province, P.R. China. *Hemoglobin* **1993**, *17*, 563–7.
65. Jiang, N.H.; Liang, S. The beta+-thalassemia mutation [IVS-II-5 (G-->C] creates an alternative splicing site in the second intervening sequence. *Hemoglobin* **1999**, *23*, 171–6.
66. Nadkarni, A.; Gorakshakar, A.; Surve, R.; Sawant, P.; Phanasgaonkar, S.; Nair, S.; Ghosh, K.; Colah, R.B. Hematological and molecular analysis of novel and rare beta-thalassemia mutations in the Indian population. *Hemoglobin* **2009**, *33*, 59–65.
67. Cheng, T.C.; Orkin, S.H.; Antonarakis, S.E.; Potter, M.J.; Sexton, J.P.; Markham, A.F.; Giardina, P.J.; Li, A.; Kazazian Jr., H.H. beta-Thalassemia in Chinese: use of in vivo RNA analysis and oligonucleotide hybridization in systematic characterization of molecular defects. *Proc Natl Acad Sci U S A* **1984**, *81*, 2821–2825.
68. Dobkin, C.; Pergolizzi, R.G.; Bahre, P.; Bank, A. Abnormal splice in a mutant human beta-globin gene not at the site of a mutation. *Proc Natl Acad Sci U S A* **1983**, *80*, 1184–1188.
69. Dobkin, C.; Bank, A. Reversibility of IVS 2 missplicing in a mutant human ??-globin gene. *J. Biol. Chem.* **1985**, *260*, 16332–16337.
70. Agouti, I.; Bennani, M.; Ahmed, A.; Barakat, A.; Mohamed, K.; Badens, C. Thalassemia intermedia due to a novel mutation in the second intervening sequence of the beta-globin gene. *Hemoglobin* **2007**, *31*, 433–8.
71. Nuntakarn, L.; Fucharoen, S.; Fucharoen, G.; Sanchaisuriya, K.; Jetsrisuparb, A.; Wiangnon, S. Molecular, hematological and clinical aspects of thalassemia major and thalassemia intermedia associated with Hb E-beta-thalassemia in Northeast Thailand. *Blood Cells. Mol. Dis.* **2009**, *42*, 32–5.
72. Varawalla, N.Y.; Old, J.M.; Weatherall, D.J. Rare beta-thalassaemia mutations in Asian indians. *Br. J. Haematol.* **1991**, *79*, 640–4.
73. Beldjord, C.; Lapoumeroulie, C.; Pagnier, J.; Benabadji, M.; Krishnamoorthy, R.; Labie, D.; Bank, A. A novel beta thalassemia gene with a single base mutation in the conserved polypyrimidine sequence at the 3′ end of IVS 2. *Nucleic Acids Res.* **1988**, *16*, 4927–35.
74. Cross, T.J.S.; Berry, P.A.; Akbar, N.; Wendon, J.; Thein, S.L.; Harrison, P.M. Sickle liver disease--an unusual presentation in a compound heterozygote for HbS and a novel beta-thalassemia mutation. *Am. J. Hematol.* **2007**, *82*, 852–4.
75. Rosatelli, M.C.; Pischedda, A.; Meloni, A.; Saba, L.; Pomo, A.; Travi, M.; Fattore, S.; Cao, A. Homozygous beta-thalassaemia resulting in the beta-thalassaemia carrier state phenotype. *Br. J. Haematol.* **1994**, *88*, 562–5.
76. Murru, S.; Loudianos, G.; Deiana, M.; Camaschella, C.; Sciarratta, G. V; Agosti, S.; Parodi, M.I.; Cerruti, P.; Cao, A.; Pirastu, M. Molecular characterization of beta-thalassemia intermedia in patients of Italian descent and identification of three novel beta-thalassemia mutations. *Blood* **1991**, *77*, 1342–7.
77. Gonzalez-Redondo, J.M.; Stoming, T.A.; Lanclos, K.D.; Gu, Y.C.; Kutlar, A.; Kutlar, F.; Nakatsuji, T.; Deng, B.; Han, I.S.; McKie, V.C. Clinical and genetic heterogeneity in black patients with homozygous beta-thalassemia from the southeastern United States. *Blood* **1988**, *72*, 1007–14.
78. Hattori, Y.; Yamamoto, K.; Yamashiro, Y.; Ohba, Y.; Miyamura, S.; Yamamoto, K.; Matsuno, Y.; Morishita, M.; Miyaji, T.; Era, T. Three beta-thalassemia mutations in the Japanese: IVS-II-1 (G----A), IVS-II-848 (C----G), and codon 90 (GAG----TAG). *Hemoglobin* **1992**, *16*, 93–7.
79. Jankovic, L.; Dimovski, A.J.; Kollia, P.; Karageorga, M.; Loukopoulos, D.; Huisman, T.H. A C----G mutation at nt position 6 3′ to the terminating codon may be the cause of a silent beta-thalassemia. *Int. J. Hematol.* **1991**, *54*, 289–93.
80. Vinciguerra, M.; Passarello, C.; Cassarà, F.; Leto, F.; Cannata, M.; Calvaruso, G.; Di Maggio, R.; Renda, D.; Maggio, A.; Giambona, A. Co-heredity of silent CAP + 1570 T>C (HBB: C*96T>C) defect and severe β-thal mutation: A cause of mild β-thalassemia intermedia. *Int. J. Lab. Hematol.* **2016**, *38*, 17–26.
81. Rund, D.; Dowling, C.; Najjar, K.; Rachmilewitz, E.A.; Kazazian, H.H.; Oppenheim, A. Two mutations in the beta-globin polyadenylylation signal reveal extended transcripts and new RNA polyadenylylation sites. *Proc. Natl. Acad. Sci. U. S. A.* **1992**, *89*, 4324–8.
82. Ma, S.K.; Lee, A.C.; Chan, A.Y.; Chan, L.C. A novel AATAAA-->CATAAA mutation at the polyadenylation site of the beta-globin gene. *Br. J. Haematol.* **2001**, *115*, 230–1.
83. Waye, J.S.; Eng, B.; Patterson, M.; Reis, M.D.; Macdonald, D.; Chui, D.H. Novel beta-thalassemia mutation in a beta-thalassemia intermedia patient. *Hemoglobin* **2001**, *25*, 103–5.
84. Kimberland, M.L.; Boehm, C.D.; Kazazian, H.H. Two novel beta-thalassemia alleles: poly A signal (AATAAA-->AAAA) and -92 C-->T. *Hum. Mutat.* **1995**, *5*, 275–6.

85. Orkin, S.H.; Cheng, T.C.; Antonarakis, S.E.; Kazazian, H.H. Thalassemia due to a mutation in the cleavage-polyadenylation signal of the human beta-globin gene. *EMBO J.* **1985**, *4*, 453–6.
86. Jacquette, A.; Le Roux, G.; Lacombe, C.; Goossens, M.; Pissard, S. Compound heterozygosity for two new mutations in the beta-globin gene [codon 9 (+TA) and polyadenylation site (AATAAA-->AAAAAA)] leads to thalassemia intermedia in a Tunisian patient. *Hemoglobin* **2004**, *28*, 243–8.
87. Henderson, S.J.; Timbs, A.T.; McCarthy, J.; Gallienne, A.E.; Proven, M.; Rugless, M.J.; Lopez, H.; Eglinton, J.; Dziedzic, D.; Beardsall, M.; et al. Ten Years of Routine α- and β-Globin Gene Sequencing in UK Hemoglobinopathy Referrals Reveals 60 Novel Mutations. *Hemoglobin* **2016**, *40*, 75–84.
88. Jankovic, L.; Efremov, G.D.; Petkov, G.; Kattamis, C.; George, E.; Yang, K.G.; Stoming, T.A.; Huisman, T.H. Two novel polyadenylation mutations leading to beta(+)-thalassemia. *Br. J. Haematol.* **1990**, *75*, 122–6.
89. Henderson, S.J.; Timbs, A.T.; McCarthy, J.; Gallienne, A.E.; Proven, M.; Rugless, M.J.; Lopez, H.; Eglinton, J.; Dziedzic, D.; Beardsall, M.; et al. Ten Years of Routine α - and β -Globin Gene Sequencing in UK Hemoglobinopathy Referrals Reveals 60 Novel Mutations. *Hemoglobin* **2016**, *40*, 75–84.
90. Rund, D.; Filon, D.; Dowling, C.; Kazazian, H.H.; Rachmilewitz, E.A.; Oppenheim, A. Molecular studies of beta-thalassemia in Israel. Mutational analysis and expression studies. *Ann. N. Y. Acad. Sci.* **1990**, *612*, 98–105.
91. Herrera, M.A.; De La Fuente-Gonzalo, F.; González, F.A.; Nieto, J.M.; Dominguez, A.B.; Villegas, A.; Ropero, P. Identification of a novel mutation in the β-globin gene 3′ untranslated region (HBB: c.*+118A > G) in Spain. *Hemoglobin* **2015**, *39*, 30–5.
92. Smith, D.L.; Mitui, M.; Park, J.Y.; Luu, H.S.; Timmons, C.F. Characterization of the HBB: c.*233G > C Variant: No Evidence of a β-Thalassemic Phenotype. *Hemoglobin* **2016**, *40*, 25–8.
93. Kountouris, P.; Lederer, C.W.; Fanis, P.; Feleki, X.; Old, J.; Kleanthous, M. IthaGenes: An interactive database for haemoglobin variations and epidemiology. *PLoS One* **2014**, *9*, e103020.

© 2019 by the authors. Submitted for possible open access publication under the terms and conditions of the Creative Commons Attribution (CC BY) license (http://creativecommons.org/licenses/by/4.0/).
